# Clinical characteristics of patients with *P4HTM* variant-associated epilepsy and therapeutic exploration: a case report and literature review

**DOI:** 10.3389/fneur.2024.1428076

**Published:** 2024-11-08

**Authors:** Yan-Juan Wang, Si-Xiu Li, Wen-Guang Hu, Li-Li Zhao, Mingping Lan, Jia-Lei Chen

**Affiliations:** ^1^Department of Pediatric Neurology, Chengdu Women’s and Children’s Central Hospital, School of Medicine, University of Electronic Science and Technology of China, Chengdu, China; ^2^Institute of Electronic and Information Engineering of UESTC in Guangdong, Dongguan, China

**Keywords:** *P4HTM* gene, epilepsy, HIDEA syndrome, HIF-1α inhibitor, valproate, case report, review

## Abstract

The *P4HTM* gene encodes a transmembrane prolyl 4-hydroxylase, which is responsible for the degradation of hypoxia-inducible transcription factors (HIF) under normoxia. Clinically, biallelic *P4HTM* variants have been identified in patients with hypotonia, hypoventilation, intellectual disabilities, dysautonomia, epilepsy, and eye abnormalities (HIDEA syndrome). Seizure was one of the most prominent symptoms. However, the clinical features of patients with epilepsy associated with *P4HTM* variants remain unclear. In this report, we describe a one-month-old infant with HIDEA syndrome caused by compound heterozygous *P4HTM* variants (c.300dupG/p.Gly103Argfs*22 and c.488C > T/p.Ala163Val). The infant presented with clonic seizures of focal onset that responded well to valproate, but with profound intellectual disability and global developmental delay at the last follow-up at 3 years old. A review of the existing literature indicates that seizures in this population typically begin early in infancy, manifest in multiple types, and are relatively well controlled. Epilepsy seemed unrelated to developmental outcomes or disease progression. Valproate, which has HIF-1α inhibiting properties, may be a promising treatment avenue for this population.

## Introduction

The *P4HTM* gene (OMIM* 614584) encodes a transmembrane prolyl 4-hydroxylase (P4H-TM), which is involved in the degradation of hypoxia-inducible transcription factors (HIF) under normoxia (1). The P4H-TM protein is ubiquitously expressed during embryonic, juvenile, and adult stages, with the highest levels in the brain and eye (2). Clinically, biallelic variants in *P4HTM* have been reported to cause hypotonia, hypoventilation, impaired intellectual development, dysautonomia, epilepsy, and eye abnormalities (HIDEA syndrome, OMIM# 618493) (3–7). Seizures were observed in up to 57% (17 out of 30) of the cases (8). However, there are limited reports describing epilepsy in patients with *P4HTM* variants, and the clinical features of epilepsy in this population remain unclear.

This report presents a case of early-onset epilepsy associated with HIDEA syndrome due to *P4HTM* variants, provides a comprehensive review of the existing literature on the epileptic characteristics, and discusses potential treatment strategies.

## Case report

**Figure 1:**
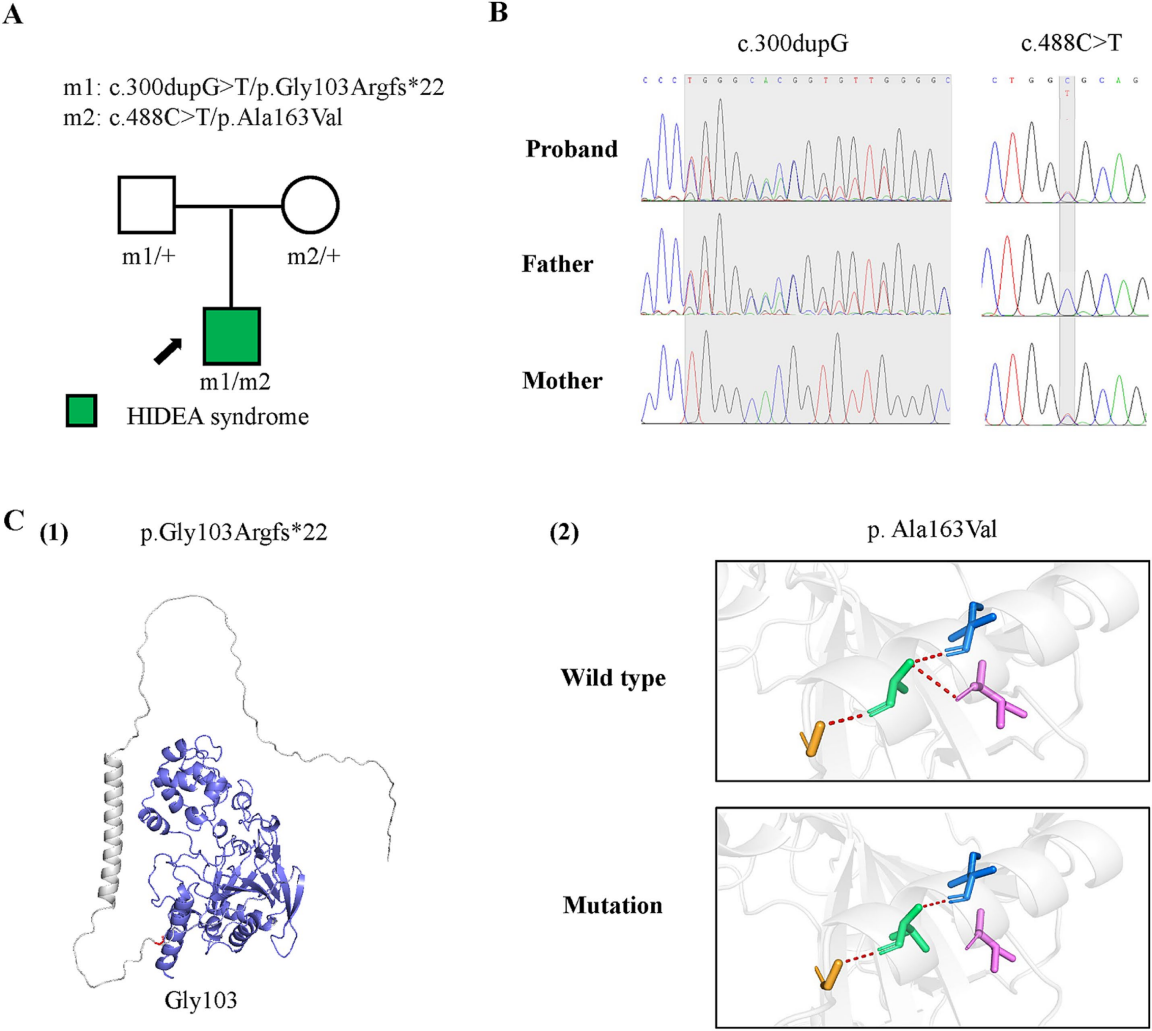
Genetic data of the case with *P4HTM* variants and molecular effect of the variants on P4H-TM protein. (A) Pedigrees of the case with *P4HTM* and their corresponding phenotypes. (B) DNA sequence chromatogram of the *P4HTM* variants. The gray regions indicate the positions of the variants. (C) Protein structure change and hydrogen bond changes of the variants from the present study. (1) The amino acid p.Gly163 is shown in red, and the deleted region in blue. (2) Hydrogen bond change of the p. Ala163Val variant.

A one-month-old male infant presented with recurrent clonic seizures, without signs of lip cyanosis, tachycardia, upward eye rolling, or drooling. A single episode of seizures lasted approximately 50 s and occurred three to five times per day. The infant was delivered at term, with a birth weight of 3.25 kg, and had a history of neonatal pneumonia. There was no family history of epilepsy or genetic disorders. Physical examination revealed hypotonia and roving eye movements (Supplementary Video S1). Laboratory tests, including full blood count, electrolytes, glucose, lactate, ammonia, liver and renal function tests, metabolic assessments, and brain magnetic resonance imaging (MRI), were all normal. Video electroencephalogram (EEG) revealed delayed maturation, multifocal discharges during both wakefulness and sleep, and captured a clonic seizure with focal onset (Figures 1A,B). According to the criteria of the Commission on Classification and Terminology of the ILAE, the infant was diagnosed with focal clonic seizures with retained awareness (9). Valproate was initiated at a dosage of 18.5 mg/kg/day for seizure control. Following subsequent titration, the infant became seizure-free at a maintenance dose of 26 mg/kg/day. At the last follow-up, the three-year-old child exhibited effective seizure control with valproate treatment, along with significant developmental delay and functioning at the level of a newborn. There was no evidence of hypoventilation, respiratory problems, obesity, or dysautonomia.

## Genetic analysis

To determine the underlying cause, genomic DNA was extracted from the proband’s and parents’ peripheral blood for trio-based whole exome sequencing. Target genes were captured by probe hybridization and enriched based on the IDT xGen Exome Research Panel. Sequence reads were aligned to the GRCh38/hg38 reference genome. Variant annotation was conducted using ANNOVAR software. Pathogenic variants were screened for their presence in exonic regions, non-synonymous mutations, and a frequency of less than 5% in databases such as ExAC, 1,000 Genomes, and gnomAD. Variants were further evaluated using databases such as dbSNP, OMIM, HGMD, and ClinVar. Sanger sequencing was used to verify variants in the proband and parents. Protein modeling was performed using AlphaFold web tool and visualization using PyMOL Molecular Graphics System 2.3.2 software.

## Genetic results

**Figure 2:**
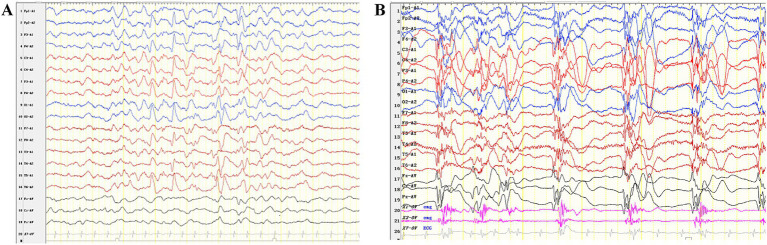
Interictal and ictal EEG recordings of the patient with *P4HTM* variants. (A) Interictal EEG of the case indicated multifocal discharges. (B) The ictal EEG shows generalized slow waves or polyspike-slow waves, along with rhythmic firing of myoelectric activity.

A pair of compound heterozygous frameshift and missense variants of the *P4HTM* gene were identified, including a paternally inherited c.300dupG/p.Gly103Argfs*22 and a maternally inherited c.488C > T/p.Ala163Val (Figures 2A,B; transcript NM_177939.3). According to the ACMG guidelines, the p.Gly103Argfs*22 variant was classified as a variant of likely pathogenic (PVSI+PM2), and the p.Ala163Val variant was classified as having uncertain significance (PM2 + PM3 + PP3) (10). However, the biallelic variants had no or less than 0.0005 frequency in the gnomAD database and had not been reported previously. The p.Gly103Argfs*22 and p.Ala163Val variants were all predicted to be deleterious by *in silico* tools and altered protein structure or hydrogen bonding with surrounding residues (Figures 1C). These results suggest that *P4HTM* is the potential pathogenic gene associated with HIDEA syndrome in this patient.

## Literature review

A literature search was conducted on PubMed and Google Scholar up to April 1, 2024, using the terms “P4HTM” or “HIDEA.” Articles describing seizures were reviewed, and a summary of the clinical and genetic profiles of the patients is presented in Table 1.

**Table 1:** Clinical features of patients with epilepsy associated with *P4HTM* variants.

	Family 1_patient 1	Family 2_patient 2	Family 2_patient 3	Family 2_patient 4	Family 2_patient 5	Family 3_patient 6	Family 4_patient 7	Family 5_patient 8	Family 5_patient 9
Variants (NM_177939.3)	c.300dupG/p.Gly103Argfs*22 & c.488C > T/p.Ala163Val (compound heterozygous)	c.1073G > A/p.Arg296Ser; Val297_Arg358del (homozygous)	c.1073G > A/p.Arg296Ser; Val297_Arg358del (homozygous)	c.1073G > A/Arg296Ser; Val297_Arg358del (homozygous)	c.1073G > A/p.Arg296Ser; Val297_Arg358del (homozygous)	c.1073G > A/p.Arg296Ser; Val297_Arg358del (homozygous)	c.286dupC/p.Gln96Profs*29 & c.482A > C/p.His161Pro (compound heterozygous)	c.1411C > T/p.Gln471* (homozygous)	c.1411C > T/p.Gln471* (homozygous)
Sex	Male	Female	Male	Female	Female	Female	Male	Male	Male
Seizures onset	One mos	After birth	2–3 mos	6 mos	22 yrs	NA	NA	NA	NA
Seizure types	Focal clonic	NA	Atonic, GTCS	Tonic	NA	NA	NA	NA	NA
Seizure frequency	5–10 times/day	NA	2–3 times/week	NA	NA	NA	NA	NA	NA
ASMs	VPA	PB, LTG	VPA + CBZ	NA	PB	NA	NA	NA	NA
ASMs response	Seizure free	NA	Seizure free	NA	Seizure free	NA	NA	NA	NA
EEG	Delayed maturation with multifocal spikes	Occipital discharges	Background abnormality, epileptiform activity	NA	NA	Slow background, ictal discharges	Epileptiform activity during sleep	NA	Multifocal spikes, δ activity
Hypotonia	Yes	Yes	Yes	Yes	Yes	Yes	Yes	Yes	Yes
ID/DD	Profound	Profound	Profound	Profound	Profound	Severe	NA	NA	Profound
Walking	No	No	Yes (4 yrs)	Yes (4 yrs)	No	Yes (2 yrs)	Yes (NA)	No	No
Language	No	No	No	No	No	Simple sentences	No speech	NA	No speech
Brain MRI	Normal	NA	NA	NA	NA	Normal	NA	NA	Ventricular and SAS enlargement
Hypoventilation	No	NA	Yes	NA	NA	Yes	Yes	Yes	Yes
Eye symptoms	No visual fixation and abnormal eye movements	Poor eye contact and alternating exotropia	Alternating exotropia	alternating exotropia	Strabismus	Alternating exotropia, vertical deviation	Intermittent alternating exotropia	Abnormal eye movements	Alternating exotropia, vertical deviation
DOT	No	Yes	No	No	No	No	Yes	No	Yes
Obesity	No	Yes	Yes	Yes	No	Yes	No	No	No
Outcome	Alive at 3 yrs	Alive at 29 yrs	Alive at 18 yrs	Alive at 55 yrs	Died at 61 yrs	Died at 5 yrs	Alive at 13 yrs	Died at 7 mos	Died at 8 yrs
Ref	This report	(3, 4)	(3, 4)	(3, 4)	(3, 4)	(3)	(3)	(3)	(3)

	Family 5_patient 10	Family 6_patient 11	Family 7_patient 12	Family 7_patient 13	Family 2_patient 14	Family 8_patient 15	Family 9_patient 16	Family 10_patient 17	Family 11_patient 18
Variants (NM_177939.3)	c.1411C > T/p.Gln471* (homozygous)	c.949delG/p.Val317fs*30 (homozygous)	c.1238C > T/p.Pro413Leu (homozygous)	c.1238C > T/p.Pro413Leu (homozygous)	c.1073G > A/p.Arg296Ser; Val297_Arg358del (homozygous)	c.1371G > A/ p.Trp457* (homozygous)	c.1073G > A/ p.Arg296Ser; Val297_ 358del &.1238C > T/p.Pro413Leu (compound heterozygous)	c.1082C > T/p.Thr361Ile (homozygous)	c.934G > A/p.Glu312Lys (homozygous)
Sex	Male	Male	Male	Male	Female	Male	Female	Male	Female
Seizures onset	NA	NA	5 mos	5 mos	NA	NA	NA	3 mos	Once at 2.5 yrs. old
Seizure types	NA	NA	Focal	NA	Focal	NA	NA	AS, focal	NA
Seizure frequency	NA	NA	NA	NA	NA	NA	NA	NA	NA
ASMs	NA	NA	NA	VPA + TPM	NA	NA	NA	Vigabatrin	PB
ASMs response	NA	NA	Seizure free	Response	NA	NA	NA	Response	Seizure free
EEG	Multifocal spikes	Focal abnormalities	NA	NA	NA	NA	NA	NA	NA
Hypotonia	Yes	Yes	Yes	Yes	Yes	Yes	No	Yes	Yes
ID/DD	Profound	Profound	Severe	Profound	Moderate	Severe	Mild	Moderate	Severe
Walking (age)	No	No	Yes (3.5 yrs)	Yes (3.5 yrs)	Yes (3 yrs)	No	Yes (1 yrs. 10 mos)	Yes (2 yrs. 4 mos)	No
Language (age)	No speech	No	Simple (4 yrs)	No	Yes (3 yrs)	No	Yes (1 yrs)	Yes (2 yrs)	Two words (3 yrs)
Brain MRI	NA	NA	Normal	NA	Normal	GBA	Normal	Normal	Normal
Hypoventilation	Yes	Yes	NA	NA	No	Yes	No	NA	NA
Eye symptoms	Abnormal eye movements	Rotating eye movements	Strabismus and exophoria	Strabismus and exophoria	Alternating exotrophia	No	Mild hyperopia	Strabismus	Strabismus
DOT	Yes	No	NA	NA	No	Yes	No	No	No
Obesity	No	NA	Yes	Yes	Yes	No	Yes	Yes	No
Outcome	Alive at 3 yrs	Died at 7 mos	Alive at 20 yrs	Alive at 12 yrs	Alive at 5 yrs. 2 mos	Alive at 3 yrs	Alive at 3 yrs. 4 mos	Alive at 6 yrs. 6 mos	Alive at 3.5 yrs
Ref	(3)	(3)	(7)	(7)	(8)	(8)	(8)	(8)	(8)

AS, asymmetrical spasm, ASMs, anti-seizure medicines; CBZ, carbamazepine; DD, developmental delay; DOT, Dysautonomia of thermoregulation; EEG, electroencephalogram; GBA, generalized brain atrophy; GTCS, generalized tonic–clonic seizure; ID, Intellectual disability; Lamotrigine LTG; Mos, months; NA, not available; PB, phenobarbital; Ref, reference; SAS, subarachnoid space; TPM, topiramate; VPA, Valproate; Yrs, years.

## Discussion

The *P4HTM* gene, located on chromosome 3p21.31, encodes P4H-TM, which is primarily expressed during the embryonic stage, particularly in the brain, emphasizing its critical role in early brain development (2). Clinically, biallelic variants in *P4HTM* gene have been reported to cause HIDEA syndrome (3–6, 8). In the present study, the infant exhibited profound global developmental delay, epilepsy, hypotonia, and abnormal eye movements after birth. Subsequently, a pair of compound heterozygous *P4HTM* variants was identified in the patient. One variant was classified as likely pathogenic, while the other was classified as of uncertain significance in accordance with the ACMG guidelines. However, the identified compound heterozygous *P4HTM* variants had no or low allele frequencies in the general population, were predicted to be damaging by *in silico* tools, and altered protein structure or affected hydrogen bonding. Based on the clinical presentations and genetic findings, the patient was diagnosed with HIDEA syndrome.

Seizure was a prominent symptom of HIDEA syndrome. A total of seven homozygous and three compound heterozygous pathogenic *P4HTM* variants were identified in 18 patients with epilepsy from 11 families (Table 1) (3, 4, 7, 8). The available data indicate that the onset of seizures varied from birth to 6 months among seven patients, with four patients exhibiting onset within the first 3 months. This finding is consistent with the predominant expression of P4H-TM in the fetal brain. However, two exceptions were noted: one patient had seizure onset at 22 years of age, while another experienced a single episode at 2.5 years. The observed seizures were of various types, including focal in four patients, generalized tonic–clonic, tonic, and atonic seizures, and epileptic spasms, each observed in one patient. Seven patients exhibited normal results, and two patients had structural abnormalities on brain MRI, including generalized brain atrophy and enlargement of the ventricles and subarachnoid space. Interictal EEG revealed background abnormalities in four cases, multifocal spikes in three cases, and focal discharges in two cases. The most frequently administered drugs were valproate and phenobarbital, each prescribed to three patients, while lamotrigine, vigabatrin, carbamazepine, and topiramate were each prescribed to one patient. Five patients received monotherapy, while two patients were treated with combination therapy including carbamazepine and valproate for one patient, and topiramate and valproate for the other. The seven cases with detailed seizure descriptions either achieved seizure freedom or responded well to anti-seizure medications.

Homozygous and heterozygous c.1073G > A variants identified in three pedigrees suggested that the c.1073G > A variant may be a hotspot variant (3, 4, 8). Despite harboring identical biallelic *P4HTM* variants (homozygous c.1073G > A, c.949delG, and heterozygous c.482A > C and c.286dupC), patients from the same family exhibited variable symptoms, with some experiencing seizures while others did not (3–5), suggesting that the disease phenotype may be influenced by additional genetic and environmental factors. The seizures observed in patients with HIDEA syndrome associated with *P4HTM* variants were effectively controlled, with no cases of drug-resistant epilepsy. These findings indicate that early-onset epilepsy induced by *P4HTM* variants cannot be classified as developmental and epileptic encephalopathy. However, despite early seizure freedom in seven cases, six patients exhibited profound or severe intellectual disability and global developmental delay, with four being nonverbal and two unable to walk. Additionally, in the population with HIDEA syndrome without epilepsy, some patients still had severe or profound intellectual disability/global developmental delay, were nonverbal, did not walk, and died in early childhood (3–6, 11). The presence or absence of seizures and their control were not correlated with either developmental outcomes or the progression of HIDEA syndrome.

In mice, homozygous deletion of p4htm results in an improper folding of the corresponding protein and overlapping phenotypic features with HIDEA patients with biallelic *P4HTM* variants, suggesting a loss-of-function disease mechanism (3, 4, 12). Thus, the compound heterozygous *P4HTM* variants identified in this patient with HIDEA syndrome may potentially be loss-of-function. Although the exact mechanism of P4H-TM in HIDEA syndrome remains unclear, it is known that P4H-TM acts on the hydroxylation of the HIF *α* protein, which is localized in the endoplasmic reticulum membrane and plays a key role in the response of cells to hypoxia (1). Knockdown of endogenous p4htm in neuroblastoma cells has been shown to increase HIF-1a protein levels in normoxia, and p4htm is induced by hypoxia in a cell type-specific manner (1). It is postulated that the disruption of HIF-1a within the endoplasmic reticulum may result in an imbalance in protein homeostasis under hypoxia, consequently triggering neuronal apoptosis. Abnormal HIF-1α levels have also been reported in some primary genetic mitochondrial diseases (13). Hay et al. observed mitochondrial dysfunction in patients with *P4HTM*-associated HIDEA syndrome and suggested that HIDEA syndrome may be a potential primary mitochondrial disorder (6).

Given the limited molecular understanding of the clinical symptoms associated with HIDEA syndrome, devising therapeutic strategies remains challenging. Nevertheless, targeting HIF-1α inhibitors may be a potential approach to prevent disease progression. Preclinical studies have demonstrated that the inhibition of HIF-1α activity has a significant impact on tumor growth (14, 15). Valproic acid, a histone deacetylase inhibitor, maintained the self-renewal of mouse embryonic stem cells under hypoxic conditions *in vitro* by suppressing HIF-1α, and downregulated the expression of HIF-1α in human retinal Müller cells under hypoxic conditions (16, 17). In patients with epilepsy associated with *P4HTM* variants, the only two individuals who received combination therapy were both treated with valproate and survived (3, 4, 7). In addition to seizure control, the present patient began valproate treatment early in life despite exhibiting profound intellectual disability and global developmental delay, as well as being nonverbal and unable to walk. Notably, the patient did not develop hypoventilation, obesity, or dysautonomia of thermoregulation, suggesting that valproate may also play a role in ameliorating other symptoms of HIDEA syndrome. Certainly, the effect of HIF-1α inhibitors including valproate in this population needs to be further confirmed.

In conclusion, the seizures observed in the patients with biallelic *P4HTM* variant-associated HIDEA syndrome are typically of infantile onset, variable in type, and relatively well controlled. Epilepsy does not appear to be associated with developmental outcome or disease progression. Valproate, which has the role of inhibiting HIF-1α, may be a promising treatment option.

## Data Availability

The datasets presented in this article are not readily available because the data are not publicly available due to privacy or ethical restrictions. Requests to access the datasets should be directed to henai6183@163.com.

## Supplementary material

Supplementary Video S1 Aberrant eye movements in patients with P4HTM variants.
