# Supplementary material for: Clinical characteristics of patients with P4HTM variant-associated epilepsy and therapeutic exploration: a case report and literature review
Source: Front Neurol. 2024 Nov 8;15:1428076. doi: 10.3389/fneur.2024.1428076 (PMC11581967; doi:10.3389/fneur.2024.1428076)
Supplement: Supplementary file 2 [file Data_Sheet_1.PDF]

CARE case report guidelines

CARE Checklist of information to include when writing a case report

(cc) BY-NC-ND

Topic	Item	Checklist item description	Reported on Line
Title	1	The diagnosis or intervention of primary focus followed by the words "case report"	Title
Key Words	2	2 to 5 key words that identify diagnoses or interventions in this case report, including "case report"	Key Words
Abstract (no references)	3a	Introduction: What is unique about this case and what does it add to the scientific literature?	Abstract
	3b	Main symptoms and/or important clinical findings	Clinical report, result
	3c	The main diagnoses, therapeutic interventions, and outcomes	Clinical report, result
	3d	Conclusion—What is the main "take-away" lesson(s) from this case?	Conclusion
Introduction	4	One or two paragraphs summarizing why this case is unique (**may include references**)	Conclusion
Patient Information	5a	De-identified patient specific information	N/A
	5b	Primary concerns and symptoms of the patient	Clinical report
	5c	Medical, family, and psycho-social history including relevant genetic information	Clinical report, result
	5d	Relevant past interventions with outcomes	Clinical report, result
Clinical Findings	6	Describe significant physical examination (PE) and important clinical findings	Clinical report
Timeline	7	Historical and current information from this episode of care organized as a timeline	N/A
Diagnostic Assessment	8a	Diagnostic testing (such as PE, laboratory testing, imaging, surveys)	Clinical report, result
	8b	Diagnostic challenges (such as access to testing, financial, or cultural)	Clinical report, result
	8c	Diagnosis (including other diagnoses considered)	Clinical report, result
	8d	Prognosis (such as staging in oncology) where applicable	Clinical report
Therapeutic Intervention	9a	Types of therapeutic intervention (such as pharmacologic, surgical, preventive, self-care)	Clinical report
	9b	Administration of therapeutic intervention (such as dosage, strength, duration)	Clinical report
	9c	Changes in therapeutic intervention (with rationale)	Clinical report
Follow-up and Outcomes	10a	Clinician and patient-assessed outcomes (if available)	Clinical report
	10b	Important follow-up diagnostic and other test results	Clinical report, result
	10c	Intervention adherence and tolerability (How was this assessed?)	N/A
	10d	Adverse and unanticipated events	N/A
Discussion	11a	A scientific discussion of the strengths AND limitations associated with this case report	Discussion, paragraph 2,3,5
	11b	Discussion of the relevant medical literature **with references**	Discussion, paragraph2,3,4,5
	11c	The scientific rationale for any conclusions (including assessment of possible causes)	Discussion, paragraph 2,3,4,5
	11d	The primary "take-away" lessons of this case report (without references) in a one paragraph conclusion	Discussion, paragraph 6
Patient Perspective	12	The patient should share their perspective in one to two paragraphs on the treatment(s) they received	N/A
Informed Consent	13	Did the patient give informed consent? Please provide if requested	Yes ☑ No ☐
